# Edible Algae Reduce Blood Pressure in Humans: A Systematic Review and Meta‐Analysis of Randomised Controlled Trials

**DOI:** 10.1111/jhn.70095

**Published:** 2025-07-28

**Authors:** Patricia Casas‐Agustench, Sandra Mínguez, Zoe Brookes, Raul Bescos

**Affiliations:** ^1^ School of Health Professions, Faculty of Health University of Plymouth Plymouth UK; ^2^ Faculty of Health Sciences Open University of Catalonia (UOC) Barcelona Spain; ^3^ Peninsula Dental School, Faculty of Health University of Plymouth Plymouth UK

**Keywords:** blood pressure, cardiovascular diseases, hypertension, metabolic syndrome, microalgae, seaweed, spirulina

## Abstract

**Background:**

Edible algae contain bioactive compounds such as peptides, fucoidan, polyphenols, potassium, omega‐3 fatty acids, and antioxidants that may benefit cardiovascular health, particularly in lowering blood pressure (BP) regulation. Certain species, including *Nori* and *Kelp*, are also rich in inorganic nitrate, known for its BP‐lowering effects. However, the relationship between algae consumption and hypertension remains controversial. This study evaluated the effects of edible algae on BP in humans, considering factors such as algae type, format, dosage, intervention duration, health status, and baseline BP.

**Methods:**

A systematic search of Medline‐Pubmed, Scopus and Cochrane databases was conducted through December 2024. Randomised controlled trials (RCTs) in adults (≥ 18 years), healthy or with a cardiometabolic condition, with interventions ≥ 4‐weeks and BP outcomes were included. Risk of bias was assessed using the Cochrane RoB 2 tool. Random‐effects meta‐analyses were conducted; heterogeneity and publication bias were assessed using statistical tests and plots. Sensitivity, subgroup, and meta‐regression analyses were conducted to explore sources of heterogeneity.

**Results:**

Twenty‐nine RCTs encompassing 1583 participants were included. Edible algae intake significantly reduced systolic BP (SBP: −2.05 mmHg; 95% CI: −3.80, −0.31; *p* = 0.022) and diastolic BP (DBP: −1.87 mmHg; 95% CI: −3.10, −0.64; *p* = 0.001). Heterogeneity was high for SBP (Q‐value: 230; *I* ^2^ = 75%; *p* < 0.001) and moderate for DBP (Q‐value: 102; *I* ^2^ = 68%; *p* < 0.001). *Spirulina* was the most effective algae, reducing SBP by −5.28 mmHg (*p* = 0.032) and DBP by −3.56 mmHg (*p* = 0.044). Dosage of algae > 3 g/day significantly lowered SBP (−3.71 mmHg; *p* = 0.004) and DBP (−3.05 mmHg; *p* = 0.022). Whole algae intake showed greater effects than extracts. Benefits were more pronounced in individuals with cardiometabolic risk. Meta‐regression found no independent association between algae dosage and SBP change, but baseline SBP significantly predicted both SBP and DBP reductions.

**Conclusion:**

Consuming over 3 g/day of whole edible algae, especially *Spirulina*, for at least 12 weeks significantly lowers BP, particularly in those with elevated levels. This suggests that edible microalgae may serve as a natural approach to managing hypertension, complementing pharmacological treatments.

## Summary

- Edible algae consumption was associated with reduced systolic and diastolic blood pressure, with greater effects seen in individuals with cardiometabolic risk.
- *Spirulina* appears to be the most effective algae, with whole algae forms providing more substantial benefits than extracts.
- Higher dosages of algae (> 3 g/day) result in greater reductions in blood pressure.

## 1 Introduction

Edible algae refer to aquatic organisms consumed in various culinary applications or as supplements. These algae are generally classified into macroalgae and microalgae. Macroalgae, commonly known as sea vegetables or seaweeds, include species such as *Wakame* or *Kombu*. Microalgae, on the other hand, include species like *Chlorella* or *Spirulina*, which can be cultivated in diverse environments, including oceans, lakes, and specialised algal cultivation systems [1]. Traditionally, edible algae have been an essential compound in Asian cuisines due to their rich nutrient profile, encompassing vitamins, minerals, proteins, nitrogen compounds, omega‐3 fatty acids, and dietary fibre [2]. Over the past two decades, however, their inclusion in global diets has significantly increased, driven by growing interest in their nutritional benefits and health‐promoting properties [3]. Reflecting this trend, seaweed farming and production have expanded nearly three‐fold, escalating from 118,000 tons in 2000 to 358,000 tons in 2019 [4].

Edible algae are available in a variety of forms; fresh, dried, or powdered, as well as in supplements, extracts and functional foods, enhancing their versatility for incorporating into various culinary and health‐related applications. Additionally, they are often prepared as infusions, sauces, and other edible forms to improve palatability and facilitate consumption [5]. Several bioactive compounds in edible algae may positively influence cardiovascular health, particularly by helping to lower blood pressure (BP) [6]. These include bioactive peptides, fucoidan, polyphenols, potassium, omega‐3 fatty acids and antioxidants, which have been associated with potential blood‐pressure lowering benefits [7]. We and others have shown that edible seaweeds, particularly *Nori* and *Kelp*, are rich in inorganic nitrate [8, 9], a dietary compound with well‐known BP‐lowering effects. This is particularly relevant given that high BP (hypertension) is the leading global risk factor for cardiovascular disease and premature mortality [10]. However, the relationship between edible algae consumption and hypertension remains controversial.

Four meta‐analyses published over the last decade have reported varying outcomes [11, 12, 13, 14] regarding the effects of edible algae consumption on BP regulation. Fallah, et al. (2018) [11] found that *Chlorella* supplementation (> 4 g/day) for at least 8 weeks significantly reduced systolic (SBP) and diastolic (DBP) BP in hypertensive participants. Conversely, the other three meta‐analyses reported significant reductions in DBP only, following several weeks of *Spirulina* [12, 14] as well as all algae [12, 13] supplementation in heterogenous cohorts, including healthy individuals and patients with type 2 diabetes, hypertension, ischaemic heart disease, dyslipidaemia, and HIV [11, 13]. Currently, it is difficult to explain these discrepancies, however, factors such as relatively small sample sizes (*n* < 500), differences in dosage, and different durations of intervention may contribute to the inconsistent findings across these previous studies. For example, systematic reviews and meta‐analyses are recommended to include a minimum of three to four studies, with a total sample size of at least 1000 participants, to ensure robust and reliably findings [15]. Furthermore, it is important to acknowledge that meta‐analysis of several small studies may not reliably predict the outcomes of larger trials.

Thus, based on these considerations, the present systematic review and meta‐analysis aimed to provide the most comprehensive evaluation to date of the effects of edible algae consumption on BP in humans, particularly in healthy individuals or those at risk of cardiometabolic disease. This analysis takes into account key factors such as type and form of algae, dosage, duration of intervention, participant's health status, and baseline BP levels. Notably, this study is unique in incorporating meta‐regression analysis, allowing for a more detailed examination of the association between edible algae dosage and changes in BP.

## 2 Methods

The planned protocol for this systematic review and meta‐analysis was developed in accordance with the Preferred Reporting Items for Systematic Reviews and Meta‐Analyses (PRISMA) guidelines [16] and is detailed in Supporting Information S1: Table S1. Before initiating data collection, the systematic review and meta‐analysis was registered with PROSPERO (Registration number: CRD42022345210).

The research question was developed using the Population, Intervention, Control, and Outcome (PICO) framework (Table 1).

**Table 1:** Study eligibility criteria based on PICO framework.

Parameter	Criterion
Participants	Adults (people aged > 18 years) healthy or with chronic pathologies
Intervention/exposure	Edible algae (and/or its extracts)
Comparator	Placebo (or control)
Outcomes (main)	Blood pressure (systolic and diastolic blood pressure)

### 2.1 Search Strategy and Eligibility Criteria

Three databases (Medline‐Pubmed, Scopus and Cochrane) were searched to identify all relevant studies from inception through to December 31, 2024, using the following keywords: (“Seaweed”[Mesh] OR “Microalgae”[Mesh] OR “Kelp”[Mesh] OR “Laminaria”[Mesh] OR “Algae” OR “Laminaria japonica” OR “Nori” OR “Wakame” OR “Undaria” [Mesh] OR “Sea mustard” OR “Sea lettuce” OR “Sea kale” OR “Nostoc” [Mesh] OR “Gelidium” OR “Hijiki” OR “Sargassum fusiforme” OR “Sargassum” [Mesh] OR “Hizikia fusiforme” OR “Gracilaria” [Mesh] OR “Ulva clathrate” OR “Ulva” [Mesh] OR “Spirulina” [Mesh] OR “Chlorella” [Mesh] OR “Algal polysaccharide” OR “Trehalose” OR “Fucoidan” OR “Brown seaweed” OR “Brown algae” OR “alginate” OR “Ecklonia cava”) AND (“Humans” [Mesh]) AND (“Blood pressure” [Mesh] OR “Hypertension” [Mesh] OR “systolic blood pressure” OR “diastolic blood pressure” OR “SBP” OR “DBP”). The detailed search strategies are provided in Supporting Information S1: Table S2.

The search was limited to interventional human studies. Eligible studies included adult individuals (aged 18 years or older), who were either healthy or had chronic conditions such as hypertension, diabetes mellitus, metabolic syndrome or overweight/obesity. Only experimental studies with a minimum intervention duration of 4 weeks were included. Additionally, studies were required to report BP outcomes and be published in English. Studies without adequate results, as well as reviews, letters, comments, abstract or those conducted in animals, were excluded.

### 2.2 Study Selection

Search results were downloaded to EndNote 20 (Clarivate Analytics, Philadelphia, PA) and duplicates were excluded. The systematic review follows a three‐step method: title screening, abstract review, and full‐text analysis. Two researchers (Patricia Casas‐Agustench and Sandra Mínguez) independently screened titles and abstracts for eligibility, with a third researcher (Raul Bescos) resolving disagreements. Full texts versions were retrieved for articles that met or appeared to meet the inclusion criteria.

### 2.3 Data Extraction

The extracted data from the eligible studies included: first author's name, year of publication, study location, sample size of both experimental and control groups, study design, intervention duration, daily dosage and type of algae and/or its extracts, participants' age and gender, health status, baseline and post‐intervention mean values for SBP and DBP with standard deviations (SDs), as well as mean changes in SBP and DBP from baseline, along with their SDs where reported.

### 2.4 Risk of Bias Assessment

The risk of bias was assessed using the Revised Cochrane risk of bias tool for randomised trials (RoB 2) [17]. The RoB 2 tool evaluates five key domains: (1) bias arising from the randomisation process, (2) bias due to deviations from intended interventions, (3) bias due to missing outcome data, (4) bias in the measurement of the outcome and (5) bias in the selection of the reported result. Each study was assigned an overall bias score and categorised as having ‘low’, ‘some concerns’, or ‘high’ risk of bias. To visualise this, the Risk‐of‐bias VISualization (robvis) web was utilised [16]. The bias assessment was independently performed by one researcher (Sandra Mínguez) and subsequently verified for accuracy by another researcher (Patricia Casas‐Agustench).

### 2.5 Meta‐Analyses

#### 2.5.1 Quantitative Data Synthesis

If the outcome measures were presented as mean with variation range or inter‐quartile range, SDs were calculated using the method described by Hozo et al. [18]. In cases where only the standard error (SE) was reported, SDs were estimated using the formula: SD = SE × √*n*, where *n* refers to the number of subjects [19]. When mean changes in SBP and DBP from baseline were not provided, BP changes were estimated by subtracting baseline values from final values. SDs for mean differences were calculated using the formula: SD = √ [(SDpre‐treatment)^2^ + (SDpost‐treatment)^2^ – (2 R × SDpre‐treatment × SDpost‐treatment)], with a correlation coefficient (*R*) of 0.5 assumed for pretreatment and posttreatment comparisons [20]. In randomised controlled parallel studies with two intervention groups and a control group, data from the intervention groups were combined and analysed to assess the effect of dosage on both SBP and DBP. Missing information not presented in tables or the main text was extracted from figures. For crossover trials, separate means and SDs were used for the intervention and control groups. This method was employed to yield a more cautious estimate of the effect size in these studies, which could result in reduced statistical power and a smaller observed effect size [21].

The statistical analysis was conducted using the Statistical Package for Social Sciences Version 28 (SPSS v28, SPSS Inc., IBM Corp, Armonk, NY, USA). Random effect models were implemented to account for heterogeneity across participant and trial characteristics. Effect sizes and 95% confidence intervals (CIs) were determined through inverse variance weighting, and results were visualised in separate forest plots for SBP and DBP. Funnel plots were used to assess publication bias, with Egger's regression intercept [22] and Trim and Fill method employed to adjust effect sizes for potentially missing studies [23]. Relative trial weightings were calculated, where substantial variation indicated heterogeneity, categorised “high” (*I* ^2^ ≥ 75%), “moderate” (*I* ^2^ between 25% and 75%), or “low” (*I* ^2^ < 25%) [24]. Statistical significance was defined as *p* ≤ 0.05 across all analyses.

Subgroup analyses were performed to evaluate the differential effects of algae supplementation, considering factors such as algae type (e.g., macro‐ and microalgae), dosage, format, baseline SBP and DBP, health status and duration of intervention. Additionally, a dose–response meta‐regression analysis was conducted to evaluate the association between edible algae dosage and BP outcomes (SBP and DBP). Analyses were performed in R (RStudio version 2024.04.2) using the metafor package with random‐effects models and restricted maximum likelihood estimation (REML). Model 1 included algae dosage as the sole predictor, while Model 2 adjusted for baseline SBP and DBP. Statistical heterogeneity was assessed using the Q‐test and *I*² statistic. Residual heterogeneity after covariate adjustment was also examined. Bubble plots were generated using ggplot2 to visualise the relationship between dose and effect size, with bubble size reflecting study precision (1/SE) and a linear trend line with 95% confidence intervals.

## 3 Results

### 3.1 Search Results

**Figure 1:**
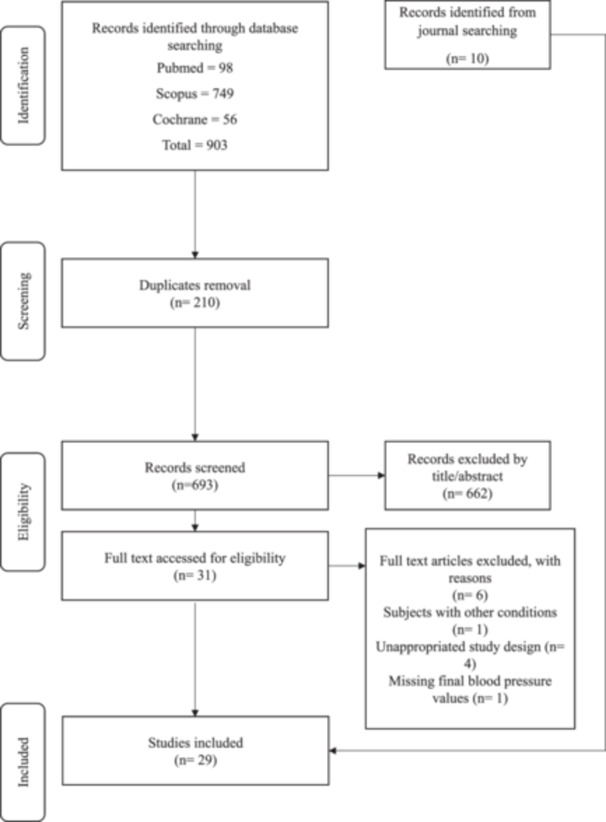
PRISMA flow diagram of the studies included in this review.

The initial database search yielded 693 unique studies (Figure 1). After screening titles and abstracts, 662 articles were excluded, followed by the exclusion of an additional 12 articles after full text review. Ten articles were identified from journal searching. Finally, 29 studies from 12 countries were included in the final analysis (Table 2).

**Table 2:** Characteristics of the studies included in the systematic review and meta‐analysis evaluating the effect of edible algae on blood pressure.

Author and year	Country	Population	Trial: Size (n),^a^ Age range (years), Female (%)	Duration (weeks)	Intervention (daily) Type of edible algae (colour)/Form of administration/Whole edible algae, extract or bioactive compound use	Baseline BP^b^ intervention group: SBP^c^, DBP^d^ (mmHg)^e^	Change in BP intervention group: SBP, DBP (mmHg)^e^	Baseline BP control group: SBP, DBP (mmHg)^e^	Change in BP control group: SBP, DBP (mmHg)^e^
Cronin, et al. 2016	Ireland	Postmenopausal	*n*: 214, y^f^: 54–67, %: 100	104.3	2.4 g Aquamin (providing 0.8 g of Ca from *Lithothamnion species*) Macroalgae (red)/NE^g^/extract	135.4 ± 21.6, 85.0 ± 10.8	−2 ± 25, −2 ± 11	131 ± 16, 85 ± 11	2 ± 18, −1 ± 11
				104.3	2.4 g Aquamin (0.8 g of Ca from *Lithothamnion species)* + 3 g scFOS^h^ (NutraFlora) Macroalgae (red)/NE/extract	137.2 ± 20.9, 86.3 ± 12.8	−0.4 ± 21, −3 ± 13	131 ± 16, 85 ± 11	2 ± 18, −1 ± 11
Ghaem Far, et al. 2021	Iran	Hypertension	*n*: 41, y: 24–65, %: 54	8.0	20 g of low‐fat salad dressing containing 2 g of *Spirulina* (*Arthrospira platensis*) powder with a vegetable salad Microalgae/powder/whole edible algae	145 ± 3, 97 ± 2	−6 ± 3, −4 ± 2	141 ± 4, 91 ± 4	0 ± 4, −1 ± 3
Hata, et al. 2001	Japan	Hypertension	*n*: 36, y: 40–86, %: 67	8.0	5 g of dried wakame (*Undaria pinnatifida*) dried powder Macroalgae (brown)/capsule/extract	158 ± 2, 90 ± 3	−8 ± 3, −8 ± 3	152 ± 3, 87 ± 2	−3 ± 3, 0 ± 3
Hernandez‐Corona, et al. 2014	Mexico	Overweight and obese	*n*: 25, y: 30–60, %: 76	13.0	0.5g F‐fucoidan Macroalgae (brown)/NE/extract	113 ± 15, 72 ± 12	−1 ± 14, −4 ± 13	119 ± 12, 78 ± 8	−3 ± 11, 1 ± 8
Hitoe, et al. 2017	Japan	Mildly obese	*n*: 27, y: 20–59, %: ND	4.0	0.001 g fucoxanthin Macroalgae (brown)/capsule/extract	125 ± 13, 73 ± 12	−4 ± 15, −3 ± 11	133 ± 10, 83 ± 11	−6 ± 13, −2 ± 11
					0.003 g fucoxanthin Macroalgae (brown)/capsule/extract	119 ± 10, 65 ± 10	−5 ± 11, 0.4 ± 9	133 ± 10, 83 ± 11	−6 ± 13, −2 ± 11
Hosseini, et al. 2021	Iran	Type 2 diabetes mellitus	*n*: 75, y: 20–65, %: 61	8.0	1.5 g *Chlorella vulgaris* powder Microalgae/capsule/extract	127 ± 12, 77 ± 6	−1 ± 12, 0 ± 12	130 ± 13, 78 ± 10	1 ± 15, 0 ± 10
Jensen, et al. 2012	Denmark	Obese	*n*: 80, y: 20–55, %: 68	12.0	Energy‐restricted diet + 0.66 g of sodium alginate derived from *Laminaria hyperborea* and *Laminaria digitata* Macroalgae (brown)/powder/extract	133 ± 2, 88 ± 1	−2 ± 2, −3 ± 2	130 ± 2, 86 ± 1	−5 ± 2, −3 ± 1
Kwak, et al. 2012	Korea	Healthy	*n*: 51, y: 29–40, %: 61	8.0	5 g of dried *Chlorella (Chlorella vulgaris)* Microalgae/tablet/extract	114 ± 17, 77 ± 12	−0.4 ± 16, 0 ± 11	118 ± 12, 82 ± 10	−0.3 ± 11, −3 ± 10
Lee, et al. 2008	Korea	Type 2 diabetes mellitus	*n*: 37, y: 49–56, %:46	12.0	8 g freeze‐dried *Spirulina* Microalgae/pill/whole edible algae	131 ± 17, 84 ± 11	−1 ± 15, −4.2 ± 9	132 ± 17, 80 ± 11	1.6 ± 18, 3 ± 11
Lee, et al. 2010	Korea	Healthy smokers	*n*: 52, y: 20–65, %: 0	6.0	6.3 g of dried *Chlorella* powder Microalgae/pill/whole edible algae	126 ± 11, 76 ± 6	−0.9 ± 10, 2 ± 6	133 ± 14, 79 ± 6	−4 ± 18, 1 ± 12
Lee, et al. 2012	Taiwan	Metabolic syndrome	*n*: 96, y: 40–62, %: 53	12.0	4.5 g *Chlorella* Microalgae/tablet/extract	131 ± 17, 81 ± 14	−4 ± 13, −5 ± 9	131 ± 17, 75 ± 11	−4 ± 13, −2 ± 10
Martinez‐Samano, et al. 2018	Mexico	Systemic arterial hypertension	*n*: 16, y: 40–65, %: 81	12.0	4.5 g *Spirulina (Arthrospira) maxima* Microalgae/NE/whole edible algae	140 ± 9, 84 ± 5	−12 ± 8, −7 ± 5	141 ± 7, 84 ± 5	−1 ± 7, 0 ± 5
Mazloomi, et al. 2022	Iran	Nonalcoholic fatty liver disease	*n*: 46, y: 18–70, %: 52	8.0	2 g *Spirulina* Microalgae/sauce/whole edible algae	130 ± 7, 88 ± 8	−3 ± 9, −2 ± 7	128 ± 10, 92 ± 6	−3 ± 8, −2 ± 6
Miczke, et al. 2016	Poland	Overweight hypertensive	*n*: 40, y: 40–60, %: 98	12.0	2 g *Spirulina maxima* (Hawaiian *Spirulina*) Microalgae/capsule/whole edible algae	149 ± 7, 85 ± 9	−6 ± 8, −6 ± 9	150 ± 7, 84 ± 9	1 ± 8, 2 ± 8
Miyazawa et al. 2013	Japan	Healthy	*n*: 12, y: 50–68, %: 42	8.0	8 g *Chlorella* Microalgae/tablet/whole edible algae	130 ± 19, 83 ± 15	−2 ± 27, −1 ± 17	112 ± 9, 74 ± 9	−2 ± 12, −2 ± 10
Moradi, et al. 2021	Iran	Ulcerative colitis	*n*: 73, y: 18–65, %: 52	8.0	1 g *Spirulina* (*Arthospira platensis*) Microalgae/capsule/whole edible algae	119 ± 9, 80 ± 6	0 ± 5, 0 ± 3	118 ± 17, 80 ± 12	1 ± 1, 0 ± 1
Neff, et al. 2011	United States	Overweight and obese	*n*: 36, y: 18–65, %: 58	19.6	5 mL of algal DHA^i^ oil containing 2 g of DHA from *Crypthecodinium cohnii* Microalgae/oil/bioactive compound	112 ± 11, 69 ± 9	3 ± 12, 1 ± 9	116 ± 10, 71 ± 7	−1 ± 8, −1 ± 5
Nishimura, et al. 2019	Japan	Healthy	*n*: 66, y: 30–70, %: 50	6.0	2 g of dried Harudori‐kombu Macroalgae (brown)/capsule/whole edible algae	128 ± 14, 81 ± 10	−2 ± 11, −2 ± 8	122 ± 16, 79 ± 12	1 ± 10, −2 ± 7
Oben, et al. 2007	United States	Overweight and obese	*n*: 52, y: 25–60, %: ND	10.0	0.001 g algae in ProAlgaZyme Microalgae/infusion/extract	152 ± 21, 69 ± 12	−10 ± 13, −7 ± 12	158 ± 24, 73 ± 15	−1 ± 12, −2 ± 10
Okada, et al. 2017	Japan	Healthy	*n*: 27, y: 24–45, %: 0	4.0	6 g *Chlorella* (*Parachlorella beijerinckii*) Microalgae/tablet/whole edible algae	134 ± 14, 86 ± 8	−2 ± 17, −6 ± 9	134 ± 14, 78 ± 10	3 ± 16, 2 ± 10
Otsuki, et al. 2013	Japan	Healthy	*n*: 14, y: 19–21, %: 0	4.0	6 g *Chlorella* powder Microalgae/tablet/whole edible algae	119 ± 8, 68 ± 4	0 ± 8, −1 ± 4	117 ± 8, 68 ± 8	1 ± 8, 0 ± 8
Otsuki, et al. 2015	Japan	Healthy	*n*: 32, y: 45–75, %: 59	4.0	6 g *Chlorella* Microalgae/tablet/whole edible algae	125 ± 17, 76 ± 8	−3 ± 17, −2 ± 11	119 ± 16, 71 ± 8	2 ± 16, 3 ± 10
Sakai, et al. 2019	Japan	Type 2 diabetes mellitus	*n*: 19, y: 30–79, %: ND	12.0	1.62 g fucoidan Macroalgae (brown)/beverage/extract	135 ± 15, 78 ± 15	1 ± 17, −1 ± 14	137 ± 18, 80 ± 15	−2 ± 17, −2 ± 11
Sanders, et al. 2006	United Kingdom	Healthy	*n*: 79, y: 18–50, %: 51	4.0	4 g refined DHA‐rich TAG j from *Schizochytrium sp*. Microalgae*/*capsule/extract	121 ± 12, 72 ± 8	−5 ± 12, 0 ± 7	120 ± 11, 74 ± 9	−2 ± 11, −2 ± 8
Shimada, et al. 2009	Japan	High‐normal blood pressure and borderline hypertension	*n*: 77, y: 37–60, %: 47	12.0	4 g GABA^k^ ‐rich *Chlorella* Microalgae/tablet/extract	142 ± 8, 90 ± 5	−7 ± 8, −4 ± 5	144 ± 8, 92 ± 4	−2 ± 7, −2 ± 4
Shin, et al. 2012	Korea	Overweight	*n*: 97, y: 19–55, %: 44	12.0	0.072 g polyphenols from *Ecklonia cava* Macroalgae (brown)/drink/extract	119 ± 13, 74 ± 10	−3 ± 12, −1 ± 9	119 ± 15, 73 ± 11	−1 ± 15, 0 ± 10
					0.144 g polyphenols from *Ecklonia cava* Macroalgae (brown)/drink/extract	119 ± 14, 73 ± 11	−4 ± 12, −2 ± 10	119 ± 15, 73 ± 11	−1 ± 15, 0 ± 10
Spiller, et al. 2003	United States	Healthy	*n*: 35, y: 35–69, %: ND	8.0	0.12 g *Haematococcus pluvialis* Microalgae/gelcap/extract with high levels of astaxanthin	118 ± 12, 76 ± 11	5 ± 13, 1 ± 11	118 ± 16, 70 ± 10	−2 ± 14, 3 ± 10
Vodouhe, et al. 2022	Canada	Overweight and obese prediabetic	*n*: 56, y: 40–67, %: 61	12.0	0.5 g *Ascophyllum nodosum* and *Fucus vesiculosus* rich in polyphenols Macroalgae (brown)/capsule/extract	119 ± 11, 75 ± 9	2 ± 12, −1 ± 9	118 ± 11, 76 ± 7	0 ± 12, −2 ± 8
Wright, et al. 2019	Australia	Obese, nondiabetic	*n*: 72, y: 18–65, %: 67	12.9	1 g of fucoidan/polyphenol extract from *Fucus vesiculosus* Macroalgae (brown)/capsule/extract that contains bioactive compounds	129 ± 15, 79 ± 11	−2 ± 10, 0 ± 12	126 ± 18, 76 ± 10	−2 ± 14, 0 ± 8

^a^ *n*, number of participants per study.

^b^ BP, blood pressure.

^c^ SBP, systolic blood pressure.

^d^ DBP, diastolic blood pressure.

^e^ y, years of age (minimum‐maximum).

^f^ Data are presented as mean ± standard deviation.

^g^ NE, non‐specified.

^h^ scFOS, short‐hain fructo‐oligosacharides.

^i^ DHA, docosahexaenoic acid.

^j^ TAG, triacylglycerol.

^k^ GABA, gamma‐aminobutyric acid.

### 3.2 Study Characteristics

Studies were conducted from 2001 to 2022 and included 1583 adults, aged 18 – 86 years, with a BMI from 21.8 to 36.5 kg/m^2^ (Table 1). The study designs included: 2 crossover [25, 26] and 27 parallel [27, 28, 29, 30, 31, 32, 33, 34, 35, 36, 37, 38, 39, 40, 41, 42, 43, 44, 45, 46, 47, 48, 49, 50, 51, 52, 53] randomised controlled trials (RCTs) and, regarding blinding, 1 trial was single‐blinded [25], 24 were double‐blinded [26, 27, 30, 31, 32, 33, 34, 35, 36, 39, 40, 41, 42, 43, 44, 45, 46, 47, 48, 49, 50, 51, 52, 53] and 1 triple‐blinded [28] and 3 did not report blinding [29, 37, 38]. Trial duration ranged from 4 to 104.3 weeks. Eight trials were tested in healthy volunteers [25, 34, 41, 44, 46, 47, 48, 51]; and the remaining trials in population with cardiometabolic disease risk conditions including healthy smokers [36], postmenopausal [27], ulcerative colitis [42], patients with T2DM [26, 32, 37], hypertension [28, 29] and systemic arterial hypertension [38], mildly obese [31], obese [33], obese nondiabetic [53], overweight and obese [30, 43, 45], overweight [50], overweight and obese prediabetic [52], overweight hypertensive [40], metabolic syndrome [35], high‐normal BP and borderline hypertension [49], and those with nonalcoholic fatty liver disease [39].

### 3.3 Type of Edible Algae and Dosage

A total of 19 studies assessed the effect of microalgae, including *Chlorella* [25, 32, 34, 35, 36, 46, 47, 49], *Spirulina* [28, 37, 38, 40, 41, 42] and others on BP [43, 45, 48, 51], whereas 10 studies evaluated the effect of macroalgae, which included brown algae such as Wakame [29], fucoidan [26, 30, 53], fucoxanthin [31], *Ecklonia cava* [50], as well as red algae such as *Lithothamnion species* [27].

Most of the studies (*n* = 9) provided edible algae in form of supplements (capsules) [29, 31, 32, 40, 42, 44, 48, 52, 53]. Eight studies provided tablets [25, 34, 35, 41, 46, 47, 49] or pills of edible algae [36, 37, 54]. Three studies provided edible algae in from of drink [26, 45, 50]. In two other studies, edible algae was provided in form of powder [33, 36] incorporated into meals [28]. One study used edible algae in form of gelcaps [51], oil [43] and sauce [39]. Three studies did not specify the form in which the supplements were administered [27, 30, 38].

Most of the studies (*n* = 12) assessed whole edible algae [25, 28, 36, 37, 38, 39, 40, 41, 42, 44, 46, 47]; while seventeen assessed the extract or bioactive compounds of edible algae [26, 27, 29, 30, 31, 32, 33, 34, 35, 43, 45, 48, 49, 50, 51, 52, 53]. The amount of edible algae consumed in the studies included in this review ranged from 0.001 to 8 g/day.

### 3.4 Blood Pressure Data

Sixteen studies measured BP at rest [25, 27, 28, 29, 30, 33, 34, 37, 38, 40, 42, 46, 49, 50, 52, 53], seven measured BP without mentioning if it was at rest [32, 39, 44, 45, 47, 48, 51], one recorded BP readings for 24 h over the course of the study [43] and five studies did not detailed the method of BP measurement [26, 31, 35, 36, 41].

Baseline SBP values spanned from 114 to 156 mmHg, while baseline DBP ranged between 68 and 94 mmHg. Nineteen studies reported both SBP and DBP decreasing with edible algae interventions [27, 28, 29, 30, 31, 33, 35, 37, 38, 39, 40, 41, 44, 45, 46, 47, 48, 49, 50].

### 3.5 Blood Pressure

The pooled effect of edible algae intake on BP (Figure S1) showed a significant reduction in SBP by −2.05 mmHg (95% CI: −3.80, −0.31 mmHg; *p* = 0.022) and a significant reduction in DBP by −1.87 mmHg (95% CI: −3.10 to −0.64 mmHg; *p* = 0.001). There was a high degree of heterogeneity between studies in SBP (Q‐value: 230; *I* ^2^ = 75%; *p* < 0.001) and moderate degree between studies in DBP (Q‐value: 102; I^2^ = 68%; *p* < 0.001).

#### 3.5.1 Subgroup Analysis

**Figure 2:**
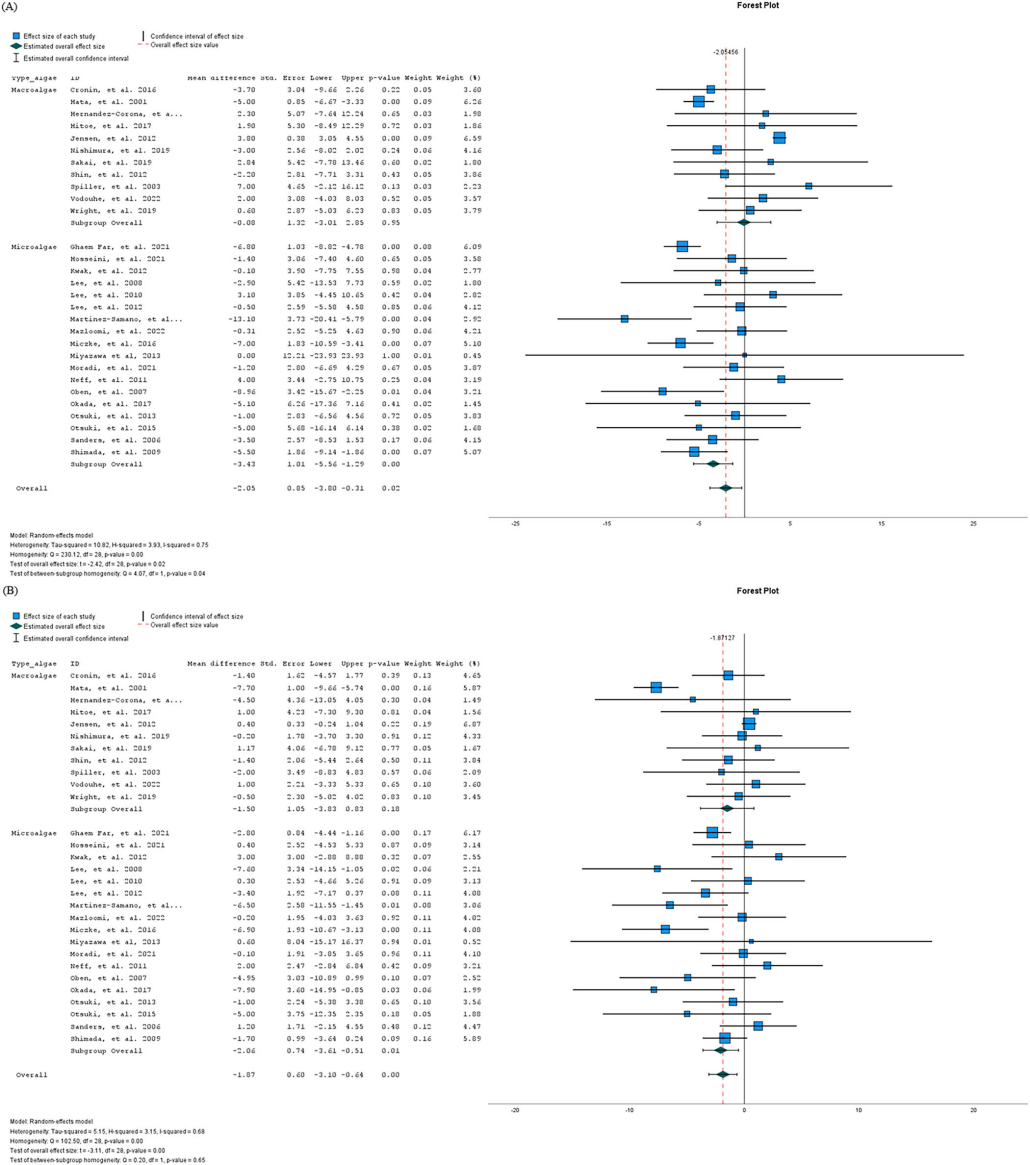
Pooled effect of edible algae on systolic blood pressure (A) and diastolic blood pressure (B) based on data from 29 randomised controlled trials by type of edible algae consumed. The squares represent mean values, with the area of each square being proportional to its relative weight in the analysis. The horizontal lines show the 95% confidence intervals; while arrows highlight cases where the lower or upper limits fall beyond −25 to +25 mmHg for systolic blood pressure and −20 to +20 mmHg for diastolic blood pressure.

Subgroup analyses were performed to explore heterogeneity among the studies. These analyses revealed a notably larger reduction in SBP by −3.43 mmHg (95% CI: −5.56, −1.29 mmHg; *p* = 0.004) and in DBP by −2.06 mmHg (95% CI: −3.62, −0.51 mmHg; *p* = 0.012) among studies using microalgae (Figure 2). No detectable effect of edible algae consumption was observed in studies consuming macroalgae for either SBP (*p* = 0.954) or DBP (*p* = 0.182).

Table 3 shows additional subgroup analyses that explore the varying effects of algae supplementation. These analyses account for factors such as the type of algae, dosage, baseline SBP and DBP, health status and duration of intervention. Those studies providing 3 or more g/day of edible algae showed a significant reduction in SBP (−3.71 mmHg; 95% CI: −5.99, −1.42 mmHg; *p* = 0.004) and in DBP (−3.05 mmHg; 95% CI: −5.57, −0.54 mmHg; *p* = 0.022), while those providing less than 3 g/day did not exhibit significant changes. *Spirulina* was the most effective edible algae for reducing SBP (−5.28 mmHg; 95% CI: −9.88, −0.67 mmHg; *p* = 0.032) and DBP (−3.56 mmHg; 95% CI: −6.97, −0.14 mmHg; *p* = 0.044), whereas other types did not show significant effects.

**Table 3:** Subgroup analysis of mean change in blood pressure.

	Change in SBP^a^ (mmHg)				Change in DBP^b^ (mmHg)			
Subgroup	Number of trials	Effect	95% CI^c^	*p* value	Number of trials	Effect	95% CI	*p* value
Health status								
Healthy	8	−1.75	−4.83, 1.25	0.207	8	−0.53	−2.87, 1.81	0.610
Cardiometabolic risk	21	−2.17	−4.30, −0.03	0.047	21	−2.12	−3.57, −0.67	0.006
Baseline SBP								
< 129 mmHg	15	−0.57	−2.43, 1.30	0.524	15	−0.03	−1.39, 1.34	0.967
≥ 129 mmHg	14	−3.55	−6.42, −0.68	0.019	14	−3.25	−5.17, −1.37	0.003
Baseline DBP								
< 79 mmHg	16	−0.03	−1.93, 1.87	0.976	16	−0.75	−2.13, 0.66	0.278
≥ 79 mmHg	13	−3.92	−6.67, −1.18	0.009	13	−2.66	−4.69, −0.64	0.014
Age								
< 46 years	13	−0.22	−2.72, 2.29	0.853	13	0.26	−0.39, 0.91	0.407
≥ 46 years	16	−3.53	−5.78, −1.28	0.004	16	−2.94	−4.67, −1.21	0.003
Type of algae								
Macroalgae: brown	9	−0.15	−3.43, 3.13	0.919	9	−1.44	−4.30, 1.42	0.279
Microalgae: *Spirulina*	6	−5.28	−9.88, −0.67	0.032	6	−3.56	−6.97, −0.14	0.044
Microalgae: *Chlorella*	9	−2.07	−4.92, 0.77	0.131	9	−1.62	−3.27, 0.03	0.053
Microalgae: others	4	−0.79	−12.0, 10.5	0.839	4	−0.21	−4.97, 4.55	0.898
Dosage								
< 3 g/day	17	−1.16	−3.56, 1.23	0.318	17	−1.07	−2.33, 0.19	0.090
≥ 3 g/day	12	−3.71	−5.99, −1.42	0.004	12	−3.05	−5.57, −0.54	0.022
Duration								
< 12 weeks	16	−2.80	−4.88, −0.73	0.012	16	−1.75	−3.65, 0.14	0.068
≥ 12 weeks	13	−1.59	−4.67, 1.50	0.284	13	−1.92	−3.70, −0.13	0.038
Whole algae versus extracts or bioactive compounds								
Whole algae	12	−3.96	−6.76, −1.16	0.010	12	−2.82	−4.80, −0.84	0.009
Extracts and bioactive compounds	17	−0.92	−3.16, 1.32	0.399	17	−1.22	−2.91, 0.46	0.143

^a^ SBP, systolic blood pressure.

^b^ DBP, diastolic blood pressure.

^c^ CI, confidence interval.

Among the differentiation between whole edible algae and extract or bioactive components use, whole edible algae intake showed a significant reduction in SBP (−3.96 mmHg; 95% CI: −6.76, −1.16 mmHg; *p* = 0.010) and in DBP (−2.82 mmHg; 95% CI: −4.80, −0.84 mmHg; *p* = 0.009), while those providing edible algae in form of extract or bioactive compounds did not exhibit significant changes.

In terms of duration, studies with interventions shorter than 12 weeks showed a positive effect for reducing SBP (−2.80 mmHg; 95% CI: −4.88, −0.73 mmHg; *p* = 0.012), while interventions longer than 12 weeks were required to see a similar effect in DBP (−1.92 mmHg; 95% CI: −3.70, −0.13 mmHg; *p* = 0.038).

A significantly greater reduction in SBP by −2.17 mmHg (95% CI: −4.30, −0.03 mmHg; *p* = 0.047) and in DBP by −2.12 mmHg (95% CI: −3.57, −0.67 mmHg; *p* = 0.006) was also identified among subjects with cardiometabolic risk. No detectable effect of edible algae consumption was observed in healthy subjects for either SBP (*p* = 0.207) or DBP (*p* = 0.610).

Subjects with a higher baseline SBP, categorised based on the mean, showed a significant greater reduction in SBP by −3.55 mmHg (95% CI: −6.42, −0.68 mmHg; *p* = 0.019) and a significantly greater reduction in DBP by −3.25 mmHg (95% CI: −5.17, −1.37 mmHg; *p* = 0.003). A similar trend was observed in participants with a higher baseline DBP, also categorised based on the mean, with significantly greater reduction in SBP by −3.92 mmHg (95% CI: −6.67, −1.18 mmHg; *p* = 0.009) and in DBP by −2.66 mmHg (95% CI: −4.69, −0.64 mmHg; *p* = 0.014).

Regarding age, subjects aged 46 years or older showed a significantly greater reduction in SBP by −3.53 mmHg (95% CI: −5.78, −1.28 mmHg; *p* = 0.004) and in DBP by −2.94 mmHg (95% CI: −4.67, −1.21 mmHg; *p* = 0.003) compared to younger individuals.

### 3.6 Meta‐Regression

The Supporting Information S1: Figure S5 illustrates the dose–response relationship between edible algae consumption and BP outcomes. No statistically significant association between edible algae dosage and changes in SBP was observed. In the univariable model (Model 1), algae dosage showed a nonsignificant trend toward SBP reduction, with an estimated effect of −0.49 mmHg per g/day (95% CI: –1.27 to 0.29; *p* = 0.216), with substantial residual heterogeneity (*I*² = 67%), suggesting that dosage alone did not account for the variability across studies. In the multivariable model (Model 2), which adjusted for baseline SBP and DBP, baseline SBP emerged as the only significant predictor of SBP reduction, with an estimated effect of −0.24 mmHg per mmHg increase in baseline SBP; 95% CI: −0.38 to −0.09; *p* = 0.002). Edible algae dosage (−0.34 mmHg per g/day; 95% CI: −1.04 to 0.36; *p* = 0.340) and baseline DBP (0.06 mmHg per mmHg; 95% CI: −0.19 to 0.30; *p* = 0.667) remained nonsignificant. Including baseline SBP reduced residual heterogeneity (*I*² = 53%), although significant between‐study variability persisted (*p* < 0.001).

For DBP, the univariable model indicated a borderline significant effect of algae dosage on DBP reduction, with an estimated effect of −0.52 mmHg per g/day (95% CI: −1.04 to 0.00; *p* = 0.051), with moderate heterogeneity (*I*² = 58%). When baseline SBP and DBP were included in the multivariable model, baseline SBP emerged as the strongest predictor of DBP reduction (−0.24 mmHg per mmHg; 95% CI: −0.32 to −0.15; *p* < 0.0001). Baseline DBP also contributed significantly, though to a lesser extent (0.14 mmHg per mmHg; 95% CI: 0.01 to 0.28; *p* = 0.033). Algae dosage became marginally significant (−0.35 mmHg per g/day; 95% CI: −0.73 to 0.02; *p* = 0.063). Notably, the inclusion of baseline values explained nearly all between‐study heterogeneity (*I*² = 6%), and residual heterogeneity was no longer statistically significant (*p* = 0.59).

### 3.7 Publication Bias

Publication bias was evaluated using a funnel plot (Supporting Information S1: Figure S2), which displayed a symmetrical distribution, indicating no evidence of bias. This visual assessment was corroborated by Egger's test, yielding nonsignificant *p* values of 0.442 for SBP and 0.927 for DBP, confirming the absence of funnel plot asymmetry. Additionally, the Trim‐and‐Fill analysis found no missing studies, with an imputed count of 0, further supporting the absence of publication bias.

### 3.8 Risk of Bias Assessment

The risk of bias assessments for the included studies are summarised in Supporting Information S1: Figure S3, with detailed information for individual studies provided in Supporting Information S1: Figure S4. Nine randomised controlled parallel studies [32] were identified as having an overall low risk of bias. However, eighteen randomised controlled parallel studies raised concerns, stemming mainly from issues such as bias arising from the randomisation process [28, 29, 30, 31, 34, 35, 36, 38, 41, 45, 50, 52], bias due to missing outcome data [27, 30, 33, 37, 43, 45], bias in outcome measurement [27, 37, 38, 42, 47], bias due to deviations from intended interventions [37, 38, 47], and bias in selecting reported results [27, 28, 30]. From crossover randomised controlled trials, both studies presented some concerns, primarily concerning the randomisation process [25, 26] or bias from the deviations in intended interventions [26].

## 4 Discussion

To the best of our knowledge, this systematic review and meta‐analysis represents the largest study to date examining the effect of edible algae on SBP and DBP, including 29 articles and data from 1,583 individuals, comprising both healthy adults or individuals with chronic cardiometabolic diseases. Our findings revealed a significant BP‐lowering effect associated with edible algae intake, particularly in people with existing high BP. Microalgaes, such as *Spirulina* also tended to cause the greatest decrease in BP. These findings align with the results from previous systematic reviews and meta‐analyses, supporting the hypotensive effect of edible algae [11, 12, 13, 14]. Additionally, unlike previous systematic reviews and meta‐analyses (Fallah et al. 2018, Arzhang et al. 2024, Ayatollahi et al. 2022, Huang et al. 2018) – of which only one included a meta‐regression with nonsignificant findings (Ayatollahi et al. 2022)‐ our study is the first to report significant results from a meta‐regression analysis. Specifically, we identified baseline SBP, rather than edible algae dosage, as the strongest predictor of BP reduction. This finding suggests that individuals with higher initial BP may experience greater benefit from edible algae consumption.

Fallah et al. (2018) [11] reported a significant reduction in SBP and DBP with *Chlorella* supplementation, while Huang et al (2018) [14] and Ayatollahi et al. 2022 [13] observed significant reductions in DBP alone with *Spirulina* supplementation and various types of edible algae, respectively. Similarly, Arzhang et al. (2024) [12] found a significant reduction in DBP with different type of edible algae, but not in SBP. However, subgroup analyses considering different types of edible algae revealed that *Spirulina* was associated with a significant decrease in SBP, whereas *Chlorella*, brown edible algae and other forms did not display significant changes.

In our study, subgroup analyses demonstrated that *Spirulina* was effective in reducing SBP and DBP. Several bioactive compounds in these algae, including peptides, carotenoids and phenolic molecules, have been associated with BP‐lowering effects [55]. Furthermore, our findings indicated that administering whole edible algae had a stronger BP‐lowering effect compared to those using isolated extracts or individual bioactive compounds. This suggests that the synergetic interaction of multiple bioactive compounds in whole edible algae provides greater benefits for reducing BP than isolating components alone.

Our analysis also revealed that consuming at least 3 g/day of edible algae may be necessary to achieve a significant reduction in BP. This finding aligns with studies conducted among elderly [29] and young [56] populations in Japan, which reported a BP lowering effect with daily intakes of edible algae. However, consuming more than 3 g/day of dried edible algae might be challenging for many individuals. Edible algae have traditionally been a dietary compound in Asian and Pacific cultures, but their consumption remains uncommon in other parts of the world. Of the 29 studies included in this review, 14 were conducted in Asian countries (9 Japan, 4 Korea, 1 Taiwan). Most of these studies used edible algae in supplement form rather than as a natural product (e.g., dried edible algae). In Japan, the average daily consumption of edible algae was estimated at 8.5 g/day in 2018 [57], slightly exceeding the typical serving size (5 g/day) for dried of edible algae [58]. In contrast, there is currently no available data on edible algae consumption in European and American diets, although it is presumed to be considerably lower than in Japan.

Notably, the reduction in SBP associated with edible algae intake was more pronounced in individuals with higher SBP baseline values (−3.55 mmHg). This finding is supported by the meta‐regression analysis, which identified baseline SBP, rather than algae dosage, as the strongest predictor of SBP response. A similar reduction in SBP (−3.7 mmHg) was reported in a meta‐analysis of 56 randomised controlled trials, which examined the effects of reducing sodium excretion by 100 mmol/day, equivalent to a daily sodium intake reduction of 2.3 g [59]. This reduction in SBP may have significant clinical implications for people with hypertension, particularly those with elevated BP. Given that hypertension is a leading risk factor for cardiovascular disease, even modest reductions in SBP can substantially lower the risk of adverse cardiovascular events such as strokes and cardiac arrest. From this point of view, another meta‐analysis of 48 randomised trials, involving 344,716 individuals, reported that a 5 mmHg reduction in SBP induced by anti‐hypertensive drugs was associated with a 10% decrease in the risk of major cardiovascular events, regardless of prior cardiovascular disease diagnoses, and even among individuals with normal or high–normal BP values [60].

Dietary changes, such as reduced sodium intake, have been shown to have an even stronger effect in reducing cardiovascular risk. An English survey spanning from 2003 to 2011, which included 9,183 individuals in 2003, 8762 individuals in 2006, 8974 individuals in 2008, and 4753 individuals in 2011 found that a 2.7 mmHg reduction in SBP, due to lower sodium intake, was associated with a 42% reduction in stroke mortality and a 40% decrease in coronary heart disease mortality [61]. Besides the mortality risk, the economic burden of stroke on public health systems is considerable. In the United Kingdom, the total cost of health and social care for acute stroke patients is estimated at £46,039/year during the first 5 years after admission [62]. Consequently, there is an urgent need for affordable and accessible interventions to manage hypertension, which would reduce the associated mortality risks and comorbidities.

On the other hand, it must be noted that sustained consumption of dried edible algae (>5 g/day) is not recommended due to potential risks, including exposure to heavy metal exposure and thyroid dysfunction [3]. The contamination of edible algae with heavy metals is closely tied to their habitat and environmental conditions. Edible algae growing in contaminated areas, often due to industrial pollution or inadequate sewage treatments, can accumulate heavy metals from nearby water sources and rocks. However, these levels are generally low and pose minimal risk to human health [63]. Nonetheless, excessive and long‐term consumption of perennial edible algae could increase the risk of heavy metal toxicity [64, 65]. Additionally, some species of edible algae are rich in iodine, which, if consumed in excess, can led to thyroid dysfunction [66]. High levels of iodine have been found in some edible macroalgae species such as *Kelp* and *Kombu* [9]. In contrast, edible microalgae contain much lower iodine levels, making them less likely to pose a risk of iodine toxicity [9]. This suggests that microalgae may offer a safer alternative for regular consumption compared to macroalgae.

Notably, microalgae stand out for their rapid growth and high reproductive rates compared to macroalgae [67]. Through the optimisation of key operational parameters and precise control of growth environments, microalgae can be cultivated effectively in large‐scale photobioreactors. This approach supports their use in diverse applications, including biofuel production, wastewater treatment, and dietary supplements [54]. Consequently, microalgae cultivation in photobioreactors offers substantial potential for sustainable industrial processes and innovative nutritional uses.

This study showcases both strengths and limitations. It offers a comprehensive assessment of edible algae's positive effect on BP, compiling findings from all pertinent clinical trials in this field. By using a pre‐registered protocol, the study guarantees a transparent and methodical approach to the meta‐analysis. Additionally, the selection of a large number of RCTs targeting a specific population aligns with the Cochrane Collaboration's stringent guidelines for systematic reviews of interventions. The search strategy stands out as a significant strength, combining digital and manual searches to comprehensively review the literature. However, there were some limitations such as the lack on the potential mechanisms that can explain the hypotensive effect of edible algae. On the other hand, one of the main limitations of the study was the high overall heterogeneity in the included studies which reduces the reliability and generalisability of the results. Additionally, the methodological approach of BP measurements could differ between the studies included in this review. Although most of the studies reported resting BP measurements; trials employing more precise BP measurement techniques, such as ambulatory BP monitoring (ABPM), are essential to better assess the effect edible algae on BP. Finally, this study did not address the potential mechanism underlying the hypotensive effects of edible algae, which limits the biological interpretation of the findings.

## 5 Conclusion

This systematic review and meta‐analysis showed that consuming more than 3 g/day of edible algae, particularly microalgae species such as *Spirulina*, for at least 12 weeks, significantly reduced SBP and DBP. This antihypertensive effect was more pronounced in individuals with elevated BP levels, suggesting that this population may particularly benefit from edible algae as a dietary intervention. Additionally, these findings highlight the potential of edible microalgae as a natural and sustainable approach to managing BP, which could complement existing pharmacological treatments. Future studies should focus on underlying the mechanisms of the BP‐lowering effect of edible algae.

## Author Contributions

Patricia Casas‐Agustench and Raul Bescos contributed to project conception and design. Patricia Casas‐Agustench and Sandra Mínguez conducted the literature search, screening, data extraction and risk of bias assessment. Patricia Casas‐Agustench and Raul Bescos statistically analysed the data. Patricia Casas‐Agustench, Zoe Brookes and Raul Bescos contributed to data interpretation. Patricia Casas‐Agustench and Raul Bescos drafted the initial paper. All authors revised and approved the final manuscript.

## Conflicts of Interest

The authors declare no conflicts of interest.

## Peer Review

The peer review history for this article is available at https://www.webofscience.com/api/gateway/wos/peer-review/10.1111/jhn.70095.

## Supporting information


**Supplementary Figure 1:** Pooled effect of edible algae on systolic blood pressure (A) and diastolic blood pressure (B) based on data from 29 randomised controlled trials.
**Supplementary Figure 2:** Funnel plot of the effect of edible algae on effect of edible algae intervention on systolic blood pressure (A) and diastolic blood pressure (B).
**Supplementary Figure 3:** Summary risk of bias per domain: randomised controlled and parallel trials (A) and randomised controlled and crossover trials (B).
**Supplementary Figure 4:** Risk of bias assessment of randomised controlled trials: (A) parallel studies and (B) crossover studies.
**Supplementary Figure 5:** Bubble plots showing the dose–response relationship between edible algae intake and blood pressure outcomes: (A) systolic blood pressure (SBP) and (B) diastolic blood pressure (DBP). Bubble size reflects study precision (1/SE), and linear trend lines with 95% confidence intervals are included.
**Supplementary Table 1:** PRISMA checklist.
**Supplementary Table 2:** Search strategies.

## Data Availability

All data pertinent to this systematic review and meta‐analysis are included in the manuscript and the Supporting materials.
