# Supplementary material for: Edible Algae Reduce Blood Pressure in Humans: A Systematic Review and Meta‐Analysis of Randomised Controlled Trials
Source: J Hum Nutr Diet. 2025 Jul 28;38(4):e70095. doi: 10.1111/jhn.70095 (PMC12304617; doi:10.1111/jhn.70095)

**Edible algae reduce blood pressure in humans: A systematic review and meta-analysis of randomised controlled trials**

Patricia Casas-Agustench, Sandra Mínguez, Zoe Brookes, Raul Bescos

| **Content** | **Caption** |
| --- | --- |
| Supplementary Table 1 (Table S1) | PRISMA checklist |
| Supplementary Table 2 (Table S2) | Search strategies |
| Supplementary Figure 1 (Figure S1) | Pooled effect of edible algae on systolic blood pressure (A) and diastolic blood pressure (B) in 29 randomised controlled trials. Squares indicate mean values, with the area of each square being proportional to its relative weight in the analysis. Horizontal lines denote 95% confidence intervals; arrows indicate a lower/upper limit outside the range of -25 mmHg to +25 mmHg for systolic blood pressure and of -20 mmHg to +20 mmHg for diastolic blood pressure. |
| Supplementary Figure 2 (Figure S2) | Funnel plot of the effect of edible algae on effect of edible algae intervention on systolic blood pressure (A) and diastolic blood pressure (B). |
| Supplementary Figure 3 (Figure S3) | Summary risk of bias per domain: randomised controlled and parallel trials (top) and randomised controlled and crossover trials (below). |
| Supplementary Figure 4 (Figure S4) | Risk of bias assessment of randomised controlled trials: (A) parallel studies and (B) crossover studies. |
| Supplementary Figure 5 (Figure S5) | Bubble plots showing the dose–response relationship between edible algae intake and blood pressure outcomes: (A) systolic blood pressure (SBP) and (B) diastolic blood pressure (DBP). Bubble size reflects study precision (1/SE), and linear trend lines with 95% confidence intervals are included. |

**Supplementary Table 1 (Table S1).** PRISMA checklist

| **Section and Topic** | **Item #** | **Checklist item** | **Location where item is reported** |
| --- | --- | --- | --- |
| **TITLE** | | |  |
| **Title** | **1** | **Identify the report as a systematic review.** | **Title page** |
| **ABSTRACT** | | |  |
| **Abstract** | **2** | **See the PRISMA 2020 for Abstracts checklist.** | **Page 1-2** |
| **INTRODUCTION** | | |  |
| **Rationale** | **3** | **Describe the rationale for the review in the context of existing knowledge.** | **Introduction, page 3-5** |
| **Objectives** | **4** | **Provide an explicit statement of the objective(s) or question(s) the review addresses.** | **Introduction, page 4-5** |
| **METHODS** | | |  |
| **Eligibility criteria** | **5** | **Specify the inclusion and exclusion criteria for the review and how studies were grouped for the syntheses.** | **Methods, page 5-6** |
| **Information sources** | **6** | **Specify all databases, registers, websites, organisations, reference lists and other sources searched or consulted to identify studies. Specify the date when each source was last searched or consulted.** | **Methods, page 5** |
| **Search strategy** | **7** | **Present the full search strategies for all databases, registers and websites, including any filters and limits used.** | **Methods, page 5-6**  **Table S2** |
| **Selection process** | **8** | **Specify the methods used to decide whether a study met the inclusion criteria of the review, including how many reviewers screened each record and each report retrieved, whether they worked independently, and if applicable, details of automation tools used in the process.** | **Methods, page 5-6** |
| **Data collection process** | **9** | **Specify the methods used to collect data from reports, including how many reviewers collected data from each report, whether they worked independently, any processes for obtaining or confirming data from study investigators, and if applicable, details of automation tools used in the process.** | **Methods, page 5-9** |
| **Data items** | **10a** | **List and define all outcomes for which data were sought. Specify whether all results that were compatible with each outcome domain in each study were sought (e.g. for all measures, time points, analyses), and if not, the methods used to decide which results to collect.** | **Methods, page 7-8** |
|  | **10b** | **List and define all other variables for which data were sought (e.g. participant and intervention characteristics, funding sources). Describe any assumptions made about any missing or unclear information.** | **Methods, page 6-7**  **Table 2** |
| **Study risk of bias assessment** | **11** | **Specify the methods used to assess risk of bias in the included studies, including details of the tool(s) used, how many reviewers assessed each study and whether they worked independently, and if applicable, details of automation tools used in the process.** | **Methods, page 7** |
| **Effect measures** | **12** | **Specify for each outcome the effect measure(s) (e.g. risk ratio, mean difference) used in the synthesis or presentation of results.** | **Methods, page 7-8** |
| **Synthesis methods** | **13a** | **Describe the processes used to decide which studies were eligible for each synthesis (e.g. tabulating the study intervention characteristics and comparing against the planned groups for each synthesis (item #5)).** | **Methods, page 5-6** |
|  | **13b** | **Describe any methods required to prepare the data for presentation or synthesis, such as handling of missing summary statistics, or data conversions.** | **Methods, page 7-8** |
|  | **13c** | **Describe any methods used to tabulate or visually display results of individual studies and syntheses.** | **Methods, page 6-9** |
|  | **13d** | **Describe any methods used to synthesize results and provide a rationale for the choice(s). If meta-analysis was performed, describe the model(s), method(s) to identify the presence and extent of statistical heterogeneity, and software package(s) used.** | **Methods, page 7-9** |
|  | **13e** | **Describe any methods used to explore possible causes of heterogeneity among study results (e.g. subgroup analysis, meta-regression).** | **Methods, page 8** |
|  | **13f** | **Describe any sensitivity analyses conducted to assess robustness of the synthesized results.** | **Methods, page 8** |
| **Reporting bias assessment** | **14** | **Describe any methods used to assess risk of bias due to missing results in a synthesis (arising from reporting biases).** | **Methods, page 6-9** |
| **Certainty assessment** | **15** | **Describe any methods used to assess certainty (or confidence) in the body of evidence for an outcome.** | **Methods, page 7-9** |
| **RESULTS** | | |  |
| **Study selection** | **16a** | **Describe the results of the search and selection process, from the number of records identified in the search to the number of studies included in the review, ideally using a flow diagram.** | **Results, page 9**  **Figure 1** |
|  | **16b** | **Cite studies that might appear to meet the inclusion criteria, but which were excluded, and explain why they were excluded.** | **Results, page 9** |
| **Study characteristics** | **17** | **Cite each included study and present its characteristics.** | **Results, page 9-11**  **Table 2** |
| **Risk of bias in studies** | **18** | **Present assessments of risk of bias for each included study.** | **Results, page 14-15** |
| **Results of individual studies** | **19** | **For all outcomes, present, for each study: (a) summary statistics for each group (where appropriate) and (b) an effect estimate and its precision (e.g. confidence/credible interval), ideally using structured tables or plots.** | **Results, page 10-15** |
| **Results of syntheses** | **20a** | **For each synthesis, briefly summarise the characteristics and risk of bias among contributing studies.** | **Results, page 14-15** |
|  | **20b** | **Present results of all statistical syntheses conducted. If meta-analysis was done, present for each the summary estimate and its precision (e.g. confidence/credible interval) and measures of statistical heterogeneity. If comparing groups, describe the direction of the effect.** | **Results, page 11-14** |
|  | **20c** | **Present results of all investigations of possible causes of heterogeneity among study results.** | **Results, page 11-14** |
|  | **20d** | **Present results of all sensitivity analyses conducted to assess the robustness of the synthesized results.** | **Results, page 11-14** |
| **Reporting biases** | **21** | **Present assessments of risk of bias due to missing results (arising from reporting biases) for each synthesis assessed.** | **Results, page 14-15** |
| **Certainty of evidence** | **22** | **Present assessments of certainty (or confidence) in the body of evidence for each outcome assessed.** | **Results, page 14** |
| **DISCUSSION** | | |  |
| **Discussion** | **23a** | **Provide a general interpretation of the results in the context of other evidence.** | **Discussion, page 15-20** |
|  | **23b** | **Discuss any limitations of the evidence included in the review.** | **Discussion, page 19** |
|  | **23c** | **Discuss any limitations of the review processes used.** | **Discussion, page 19** |
|  | **23d** | **Discuss implications of the results for practice, policy, and future research.** | **Discussion, page 16-18** |
| **OTHER INFORMATION** | | |  |
| **Registration and protocol** | **24a** | **Provide registration information for the review, including register name and registration number, or state that the review was not registered.** | **Methods, page 5** |
|  | **24b** | **Indicate where the review protocol can be accessed, or state that a protocol was not prepared.** | **Methods, page 5** |
|  | **24c** | **Describe and explain any amendments to information provided at registration or in the protocol.** | **None** |
| **Support** | **25** | **Describe sources of financial or non-financial support for the review, and the role of the funders or sponsors in the review.** | **None** |
| **Competing interests** | **26** | **Declare any competing interests of review authors.** | **Title page, none** |
| **Availability of data, code and other materials** | **27** | **Report which of the following are publicly available and where they can be found: template data collection forms; data extracted from included studies; data used for all analyses; analytic code; any other materials used in the review.** | **None** |

**Table S2.** Search strategies

| **Database** | **Search strategy** |
| --- | --- |
| Pubmed | ("Seaweed"[Mesh] OR "Microalgae"[Mesh] OR "Kelp"[Mesh] OR "Laminaria"[Mesh] OR “Algae” OR “Laminaria japonica” OR “Nori” OR “Wakame” OR “Undaria” [Mesh] OR “Sea mustard” OR “Sea lettuce” OR “Sea kale” OR “Nostoc” [Mesh] OR “Gelidium” OR “Hijiki” OR “Sargassum fusiforme” OR "Sargassum" [Mesh] OR “Hizikia fusiforme” OR “Gracilaria” [Mesh] OR “Ulva clathrate” OR "Ulva" [Mesh] OR “Spirulina” [Mesh] OR “Chlorella” [Mesh] OR “Algal polysaccharide” OR “Trehalose” OR “Fucoidan” OR “Brown seaweed” OR “Brown algae” OR “alginate” OR “Ecklonia cava”) AND ("Humans" [Mesh]) AND (“Blood pressure” [Mesh] OR “Hypertension” [Mesh] OR "systolic blood pressure" OR "diastolic blood pressure" OR "SBP" OR "DBP"). |
| Scopus | ( TITLE-ABS-KEY ( seaweed  OR  microalgae  OR  kelp  OR  laminaria  OR  algae  OR  "laminaria japonica"  OR  nori  OR  wakame  OR  undaria  OR  "sea mustard"  OR  "Sea lettuce"  OR  "Sea kale"  OR  nostoc  OR  gelidium  OR  hijiki  OR  "Sargassum fusiforme"  OR  sargassum  OR  "Hizikia fusiforme"  OR  gracilaria  OR  "Ulva clathrate"  OR  ulva  OR  spirulina  OR  chlorella  OR  "Algal polysaccharide"  OR  trehalose  OR  fucoidan  OR  "Brown seaweed"  OR  "Brown algae"  OR  alginate  OR  "Ecklonia cava" )  AND  TITLE-ABS-KEY ( "blood pressure"  OR  hypertension  OR  "systolic blood pressure"  OR  "diastolic blood pressure"  OR  sbp  OR  dbp )  AND  TITLE-ABS-KEY ( humans ) ) |
| Cochrane | 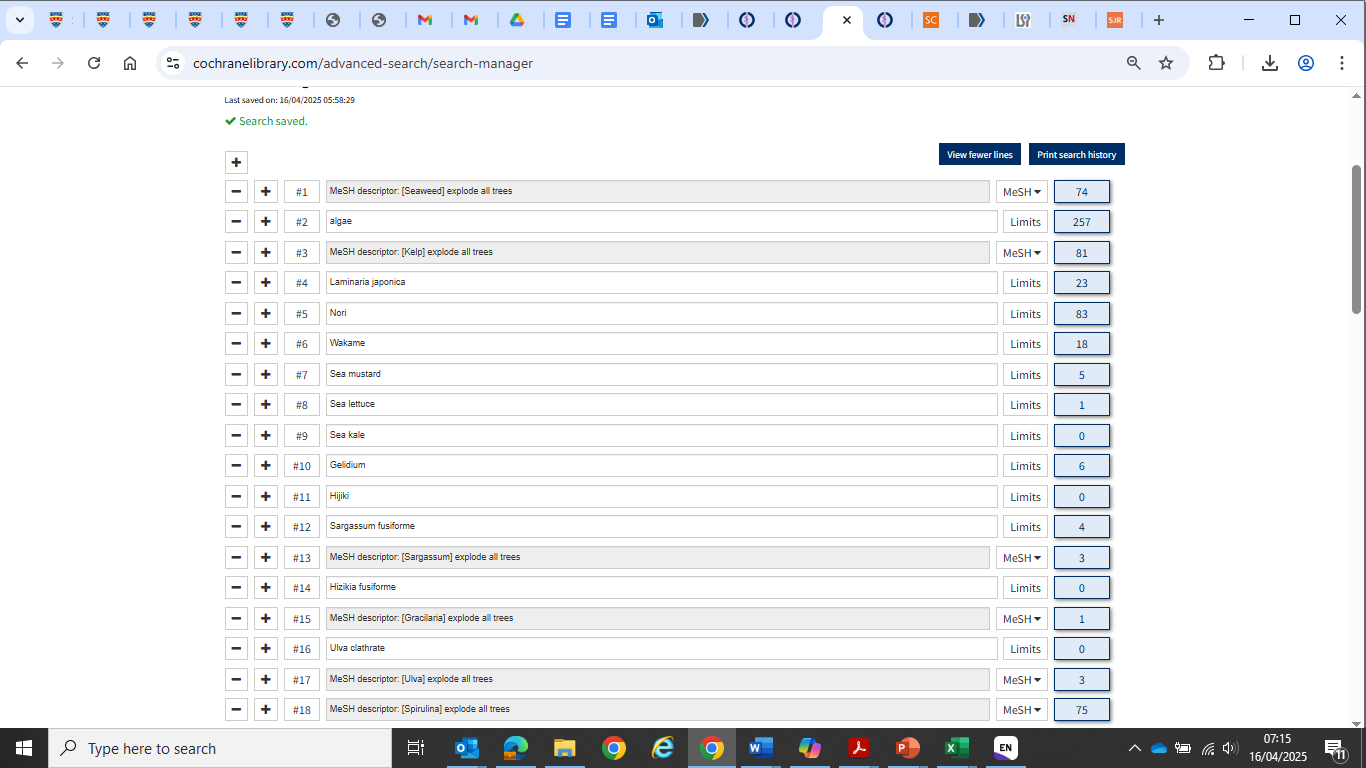  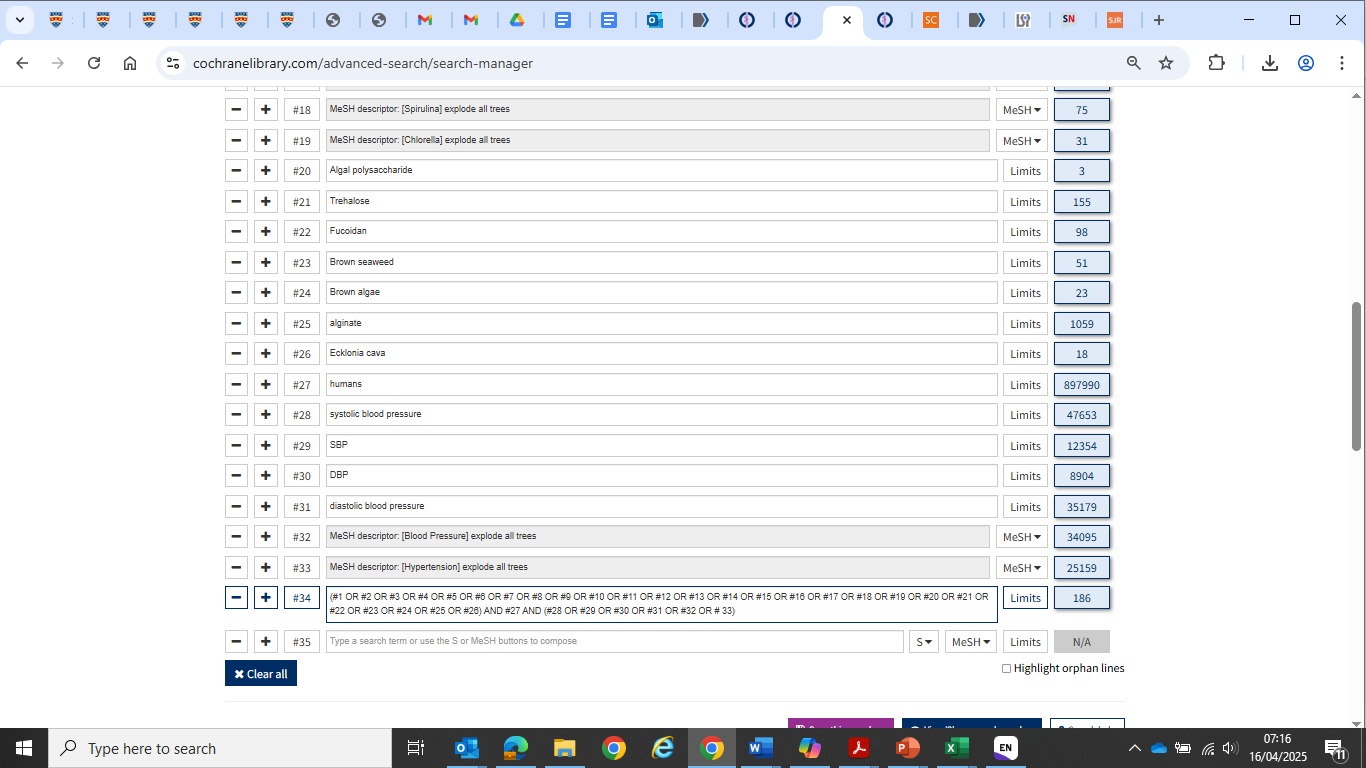 |

**Supplementary Figure 1 (Figure S1).** Pooled effect of edible algae on systolic blood pressure (A) and diastolic blood pressure (B) based on data from 29 randomised controlled trials. The squares represent mean values, with the area of each square being proportional to its relative weight in the analysis. The horizontal lines show the 95% confidence intervals; while arrows highlight cases where the lower or upper limits fall beyond -25 mmHg to +25 mmHg for systolic blood pressure and -20 mmHg to +20 mmHg for diastolic blood pressure.

(A)


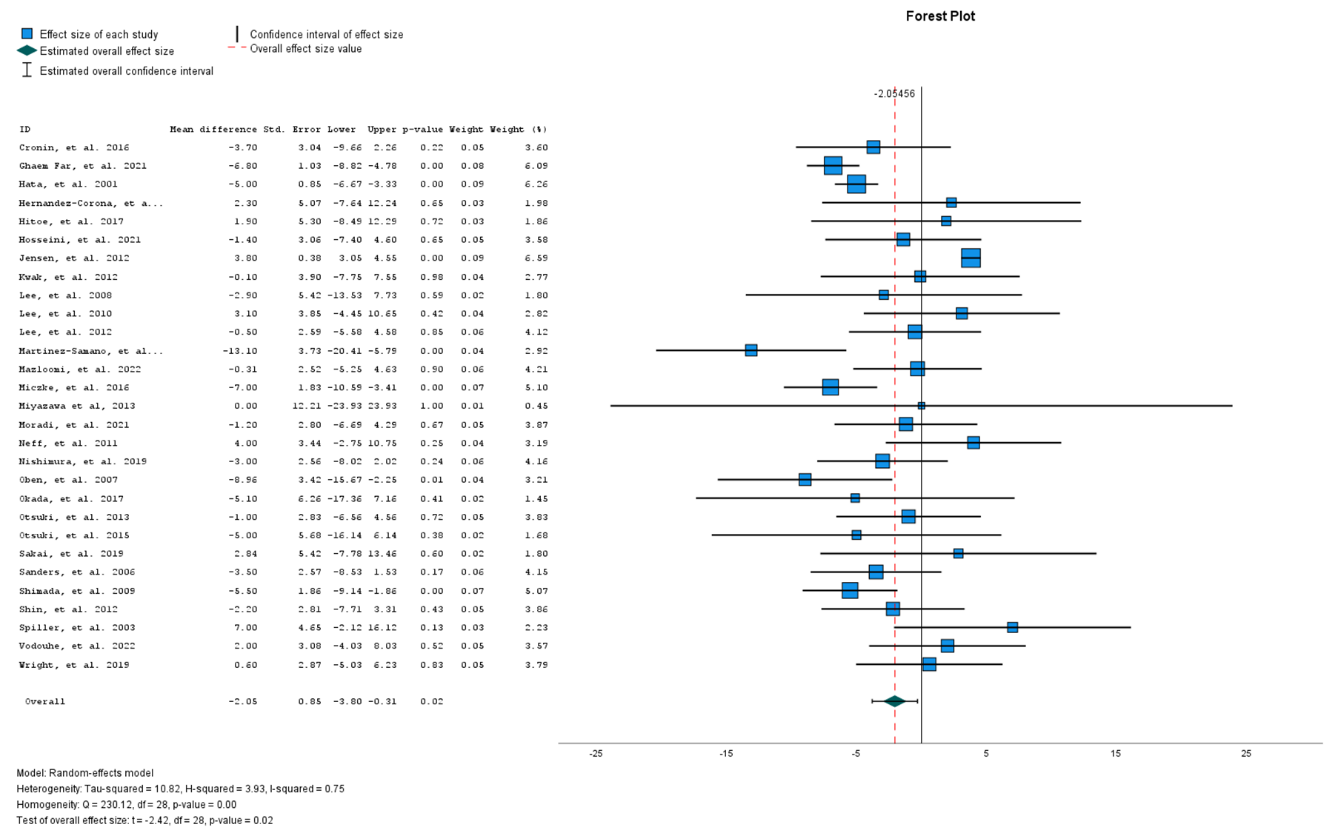


(B)


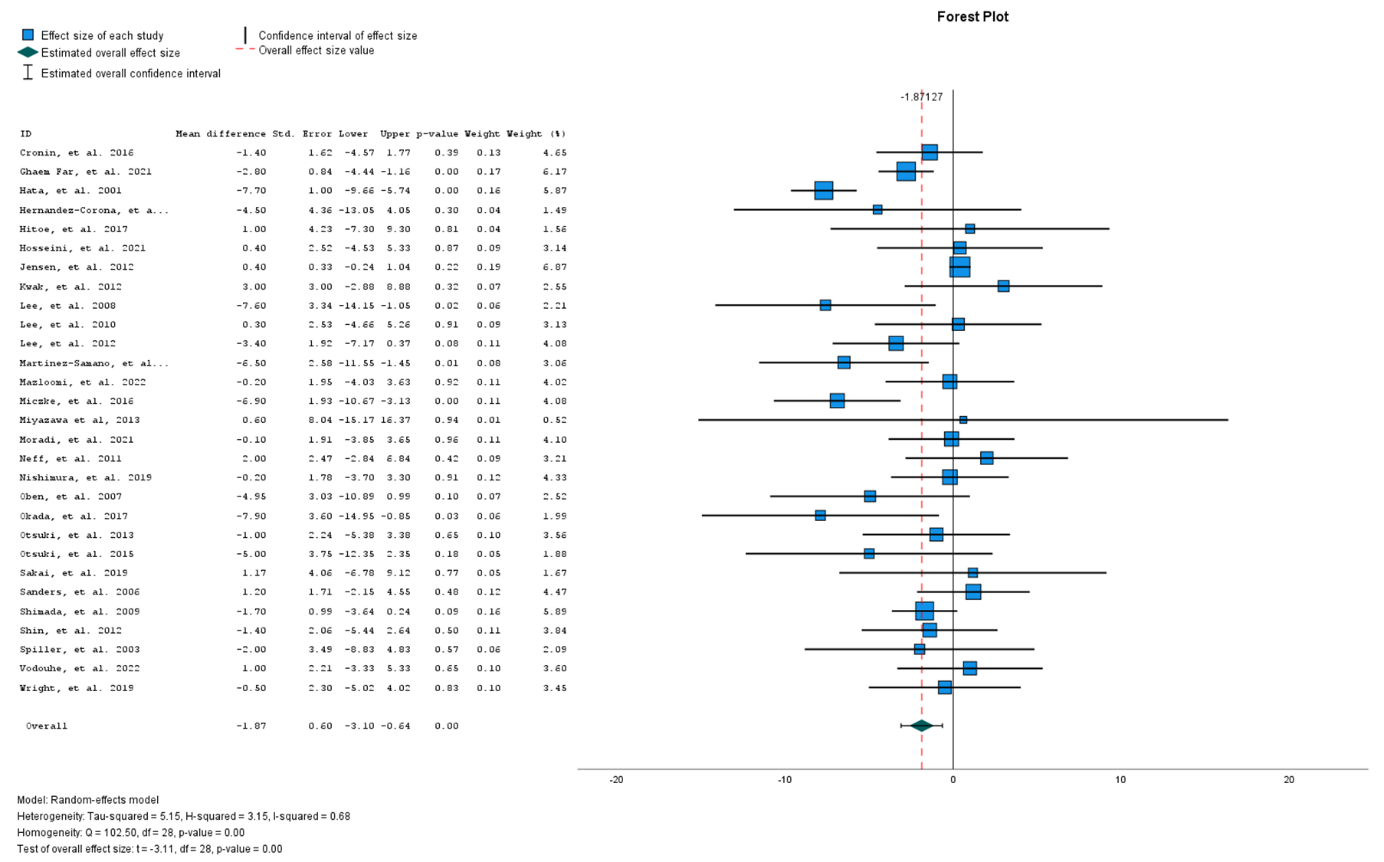


**Supplementary Figure 2 (Figure S2).** Funnel plot of the effect of edible algae on effect of edible algae intervention on systolic blood pressure (A) and diastolic blood pressure (B).

(A)


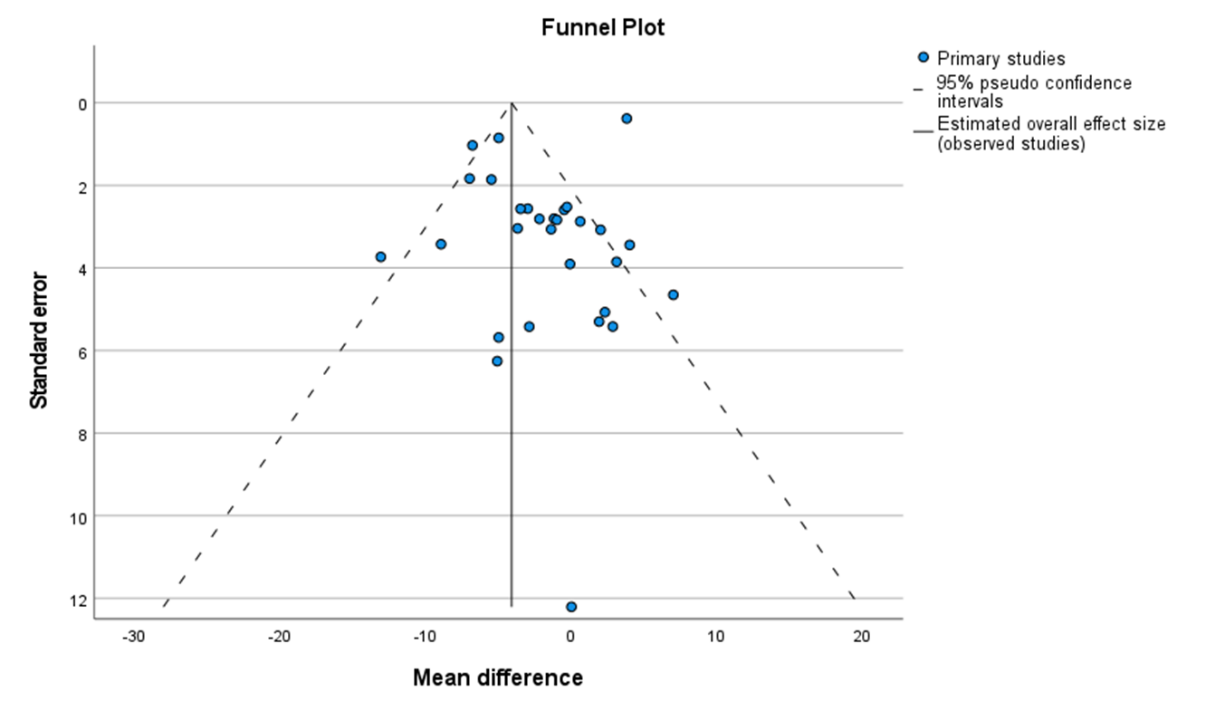


(B)


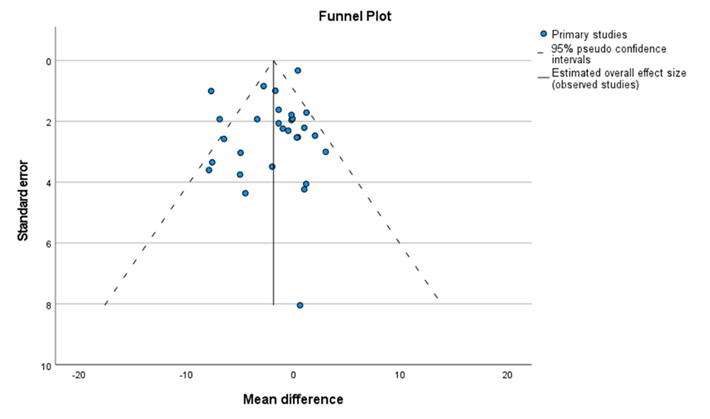


**Supplementary Figure 3 (Figure S3).** Summary risk of bias per domain: randomised controlled and parallel trials (A) and randomised controlled and crossover trials (B).


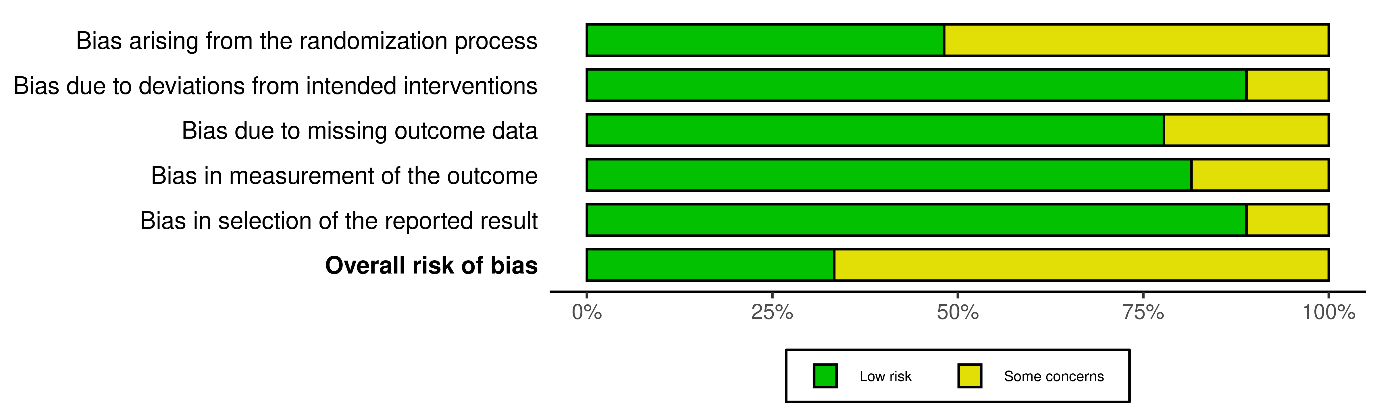


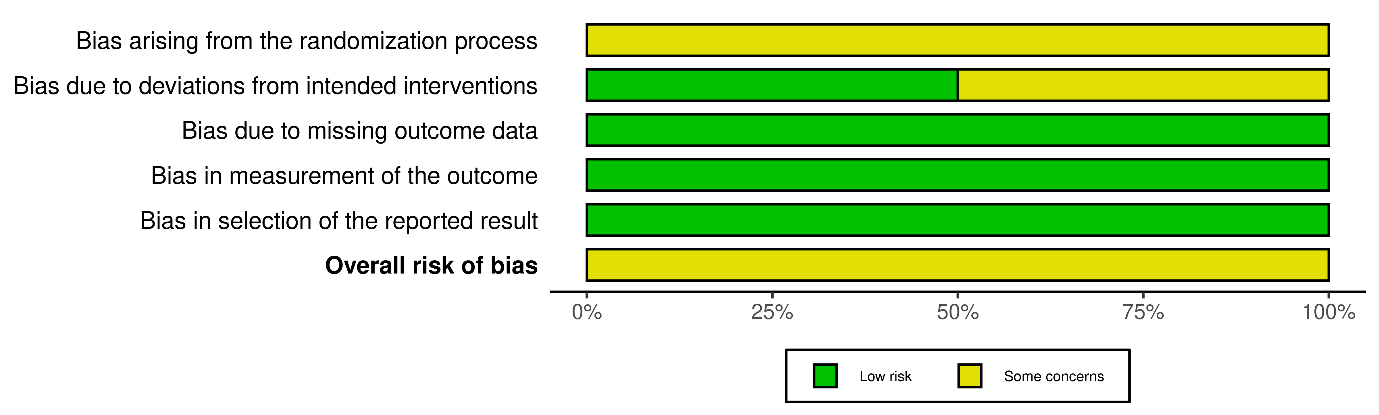


**Supplementary Figure 4 (Figure S4).** Risk of bias assessment of randomised controlled trials: (A) parallel studies and (B) crossover studies.

(A)


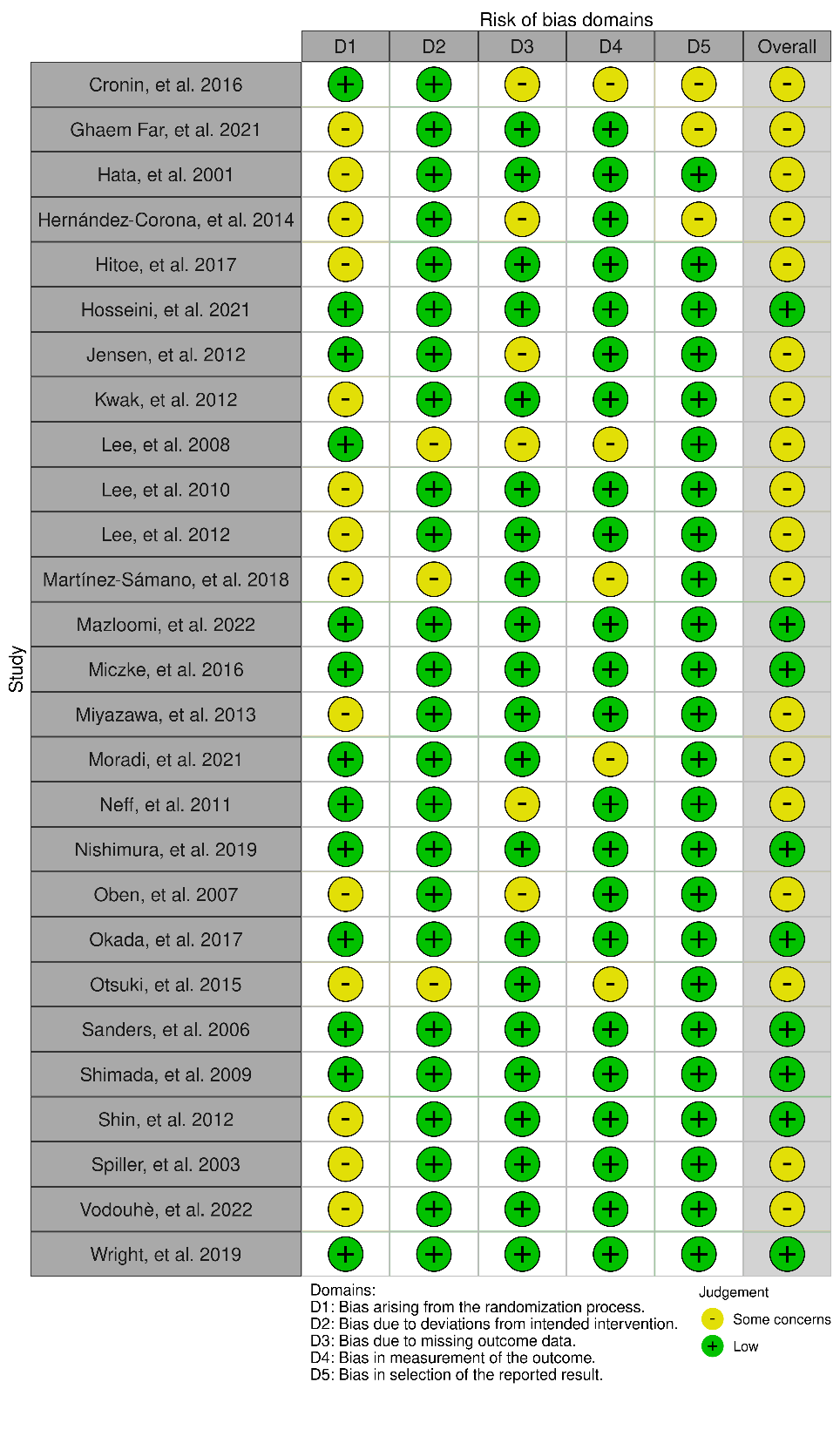


(B)

**
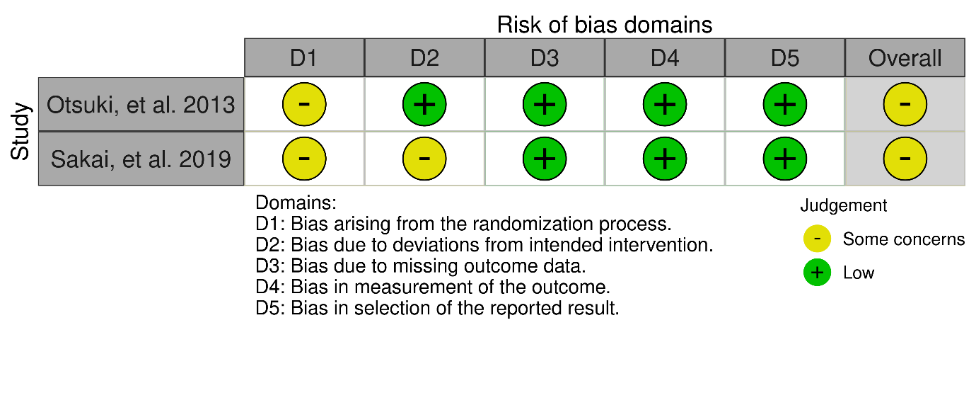
**

**Supplementary Figure 5 (Figure S5).** Bubble plots showing the dose–response relationship between edible algae intake and blood pressure outcomes: (A) systolic blood pressure (SBP) and (B) diastolic blood pressure (DBP). Bubble size reflects study precision (1/SE), and linear trend lines with 95% confidence intervals are included.

A)


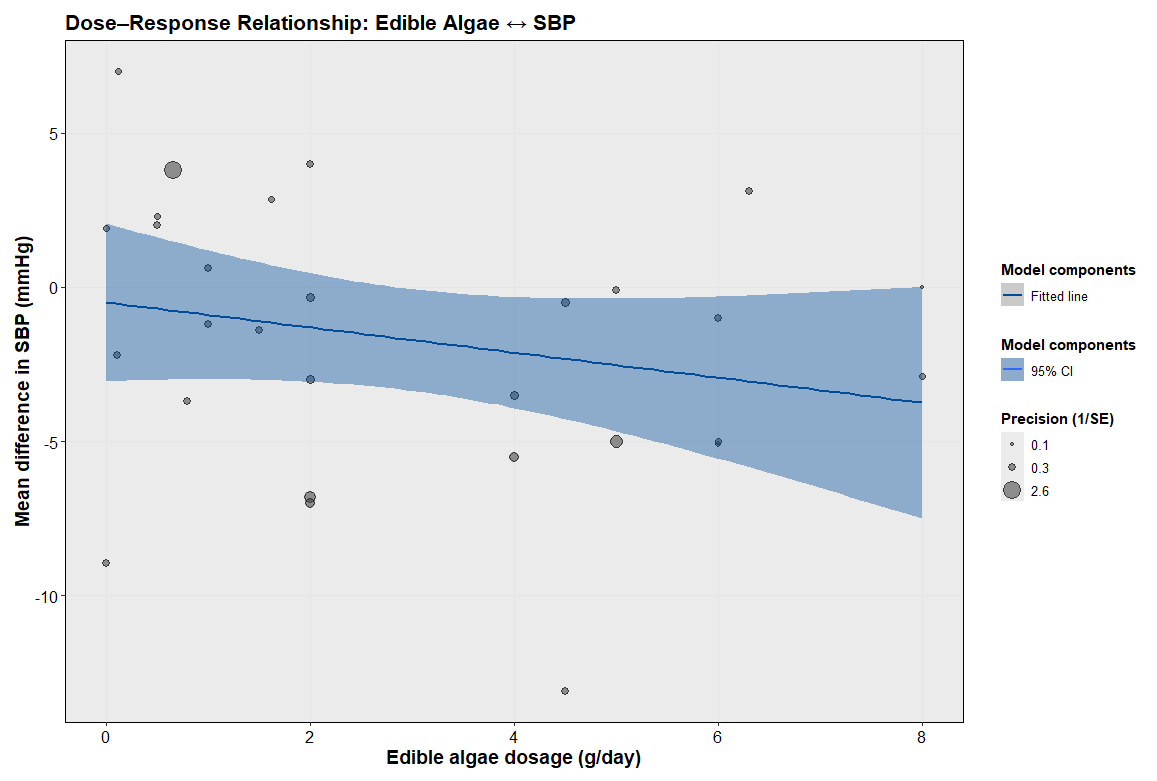


B)


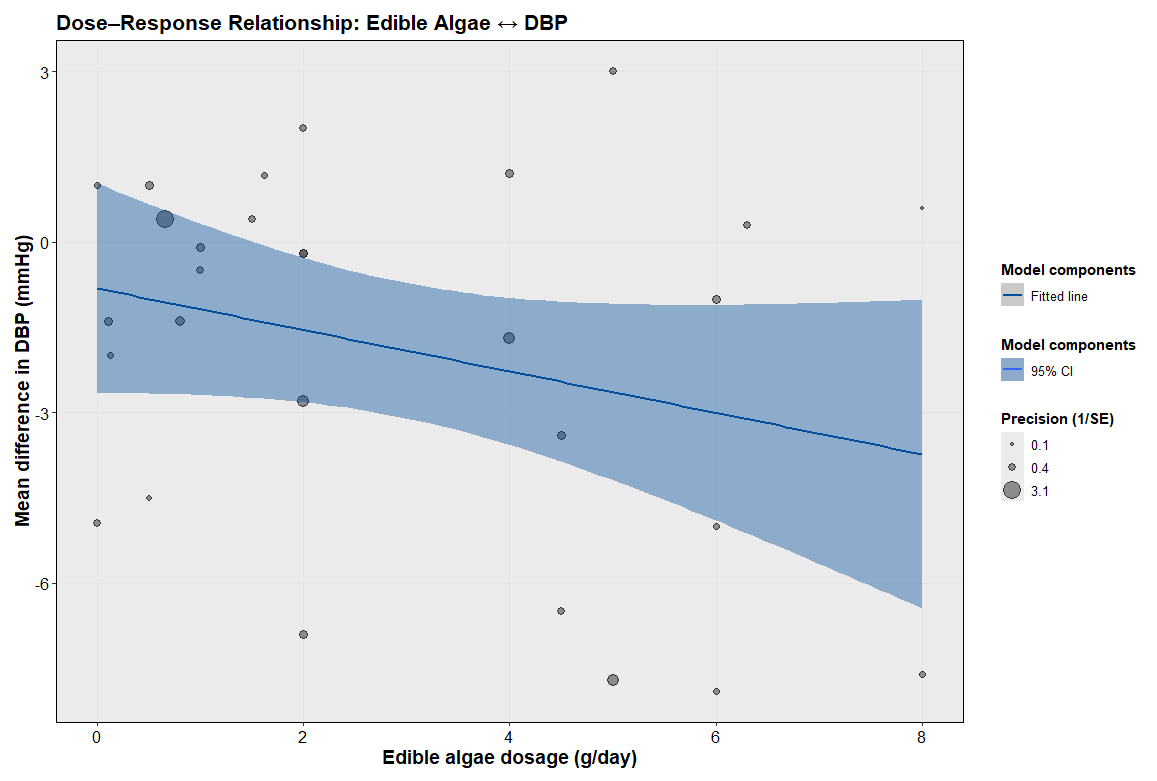

Supplement: Supplementary file 1 — Supplementary Figure 1: Pooled effect of edible algae on systolic blood pressure (A) and diastolic blood pressure (B) based on data from 29 randomised controlled trials. Supplementary Figure 2: Funnel plot of the effect of edible algae on effect of edible algae intervention on systolic blood pressure (A) and diastolic blood pressure (B). Supplementary Figure 3: Summary risk of bias per domain: randomised controlled and parallel trials (A) and randomised controlled and crossover trials (B). Supplementary Figure 4: Risk of bias assessment of randomised controlled trials: (A) parallel studies and (B) crossover studies. Supplementary Figure 5: Bubble plots showing the dose–response relationship between edible algae intake and blood pressure outcomes: (A) systolic blood pressure (SBP) and (B) diastolic blood pressure (DBP). Bubble size reflects study precision (1/SE), and linear trend lines with 95% confidence intervals are included. Supplementary Table 1: PRISMA checklist. Supplementary Table 2: Search strategies. [file JHN-38-0-s001.docx]
